# Supplementary material for: Hybrid Models and Biological Model Reduction with PyDSTool
Source: PLoS Comput Biol. 2012 Aug 9;8(8):e1002628. doi: 10.1371/journal.pcbi.1002628 (PMC3415397; doi:10.1371/journal.pcbi.1002628)
Supplement: Text S4 — Complete source code for the PyDSTool package (version 0.88.120504). Includes API documentation and help files linking to web pages. This file is identical to the current public release on Sourceforge.net. (ZIP) [file pcbi.1002628.s004.zip › PyDSTool/html/PyDSTool.Toolbox.model_primitives-module.html]

xml version="1.0" encoding="ascii"?


PyDSTool.Toolbox.model\_primitives


| Home | Trees | Indices | Help | PyDSTool |
| --- | --- | --- | --- | --- |

Package PyDSTool ::
Package Toolbox ::
Module model\_primitives

[hide private]

[frames] | no frames]

# Module model\_primitives

source code

Library of common model equation primitives.

Contains functions, expressions, etc.


|  |  |
| --- | --- |
| Variables | [hide private] |

threshold = `ModelLibrary('threshold', spectype= Fun, pars= {'k...`

tanh\_spec = `Fun('1+tanh(x/k)', ['x'], 'tanh_thresh')`

logistic\_spec = `Fun('1/(1+exp(-k*x))', ['x'], 'logistic_thresh')`

alg\_spec = `Fun('0.5*(1+x/(sqrt(k+x*x)))', ['x'], 'algebraic_th...`

linear\_decay = `ModelLibrary('linear_decay', spectype= Fun, par...`


|  |  |
| --- | --- |
| Variables Details | [hide private] |

### threshold

Value:
:   ```
    ModelLibrary('threshold', spectype= Fun, pars= {'k': [-inf, inf]}, ind
    epdomain= {'x': [-inf, inf]}, depdomain= [0, 1], description= """Smoot
    h R -> [0,1] functions of
                            one parameter that represent thresholds""")
    ```

### alg\_spec

Value:
:   ```
    Fun('0.5*(1+x/(sqrt(k+x*x)))', ['x'], 'algebraic_thresh')
    ```

### linear\_decay

Value:
:   ```
    ModelLibrary('linear_decay', spectype= Fun, pars= {'tau': [0, inf], 'x
    _inf': [-inf, inf]}, indepdomain= {'x': [-inf, inf]}, depdomain= [-inf
    , inf], description= """Linear decay given by two
                                parameters""")
    ```

  


| Home | Trees | Indices | Help | PyDSTool |
| --- | --- | --- | --- | --- |

|  |  |
| --- | --- |
| Generated by Epydoc 3.0.1 on Fri May 4 15:24:04 2012 | http://epydoc.sourceforge.net |
